# Convergent Findings of Altered Functional and Structural Brain Connectivity in Individuals with High Functioning Autism: A Multimodal MRI Study

**DOI:** 10.1371/journal.pone.0067329

**Published:** 2013-06-18

**Authors:** Sophia Mueller, Daniel Keeser, Andrea C. Samson, Valerie Kirsch, Janusch Blautzik, Michel Grothe, Okan Erat, Michael Hegenloh, Ute Coates, Maximilian F. Reiser, Kristina Hennig-Fast, Thomas Meindl

**Affiliations:** 1 Institute of Clinical Radiology, Ludwig-Maximilians University Munich, Munich, Germany; 2 Department of Psychiatry and Psychotherapy, Ludwig-Maximilians University Munich, Munich, Germany; 3 Department of Psychology, Stanford University, Stanford, California, United States of America; 4 Department of Neurology, Ludwig-Maximilians University Munich, Munich, Germany; 5 German Center for Vertigo and Balance Disorders, Ludwig-Maximilians University Munich, Munich, Germany; 6 Department of Psychiatry, University of Rostock, Rostock, Germany; 7 Department of Psychology, Ludwig-Maximilians University Munich, Munich, Germany; Centre Hospitalier Universitaire Vaudois Lausanne - CHUV, UNIL, Switzerland

## Abstract

Brain tissue changes in autism spectrum disorders seem to be rather subtle and widespread than anatomically distinct. Therefore a multimodal, whole brain imaging technique appears to be an appropriate approach to investigate whether alterations in white and gray matter integrity relate to consistent changes in functional resting state connectivity in individuals with high functioning autism (HFA). We applied diffusion tensor imaging (DTI), voxel-based morphometry (VBM) and resting state functional connectivity magnetic resonance imaging (fcMRI) to assess differences in brain structure and function between 12 individuals with HFA (mean age 35.5, SD 11.4, 9 male) and 12 healthy controls (mean age 33.3, SD 9.0, 8 male). Psychological measures of empathy and emotionality were obtained and correlated with the most significant DTI, VBM and fcMRI findings. We found three regions of convergent structural and functional differences between HFA participants and controls. The right temporo-parietal junction area and the left frontal lobe showed decreased fractional anisotropy (FA) values along with decreased functional connectivity and a trend towards decreased gray matter volume. The bilateral superior temporal gyrus displayed significantly decreased functional connectivity that was accompanied by the strongest trend of gray matter volume decrease in the temporal lobe of HFA individuals. FA decrease in the right temporo-parietal region was correlated with psychological measurements of decreased emotionality. In conclusion, our results indicate common sites of structural and functional alterations in higher order association cortex areas and may therefore provide multimodal imaging support to the long-standing hypothesis of autism as a disorder of impaired higher-order multisensory integration.

## Introduction

Autism spectrum disorders (ASD) are neurodevelopmental and behaviorally defined disorders characterized by deficits in social reciprocity, impaired communication and repetitive patterns of behavior and interests [1]. Individuals with ASD display a broad clinical variability and heterogeneity. Given that even symptomatically similar sub-entities of ASD like high functioning autism (HFA) and Asperger’s syndrome display differential neuropsychological test profiles [2] and differential patterns of structural brain alterations [3], it is difficult to derive specific conclusions from the study of heterogeneous ASD samples. Therefore the present study was confined to individuals with Asperger’s syndrome. In contrast to other variants of HFA, Asperger’s syndrome is characterized by timely language development and normal verbal IQ. Since Asperger’s syndrome and HFA will be merged into one diagnostic entity in the upcoming fifth version of the Diagnostic and Statistical Manual of Mental Disorders (DSM-V), we will refer to our sample as HFA.

The precise pathophysiology of ASD remains unclear apart from ASD that occur secondary to clearly defined neurological or genetic disorders like tuberous sclerosis or Rett syndrome. So far, genetic [4], endocrine [5] and neuroimmune [6] factors have been associated with the pathogenesis of ASD. Multiple lines of investigation suggest that these and other factors result in altered myelin development in autism [7]. As white matter myelinated axons provide communication between different gray matter sites, disruptions in their integrity may also lead to gray matter alterations and changes in functional connectivity.

These altered neural tissue properties have been proposed to be exponentially distributed meaning that early brain abnormalities in ASD increasingly affect additional regions and functional systems throughout development [8]. Therefore brain differences in ASD are likely to be subtle, complex and widely distributed rather than anatomically circumscribed. This might explain why the examination of alterations of distinct brain structures in ASD such as the cerebellum [9], the amygdala [10], the hippocampus [11] and the corpus callosum [12] so far failed to reveal a robust imaging biomarker of ASD, e.g. for association with risk genes, such as the hippocampal volume has become for Alzheimer’s disease [13].

A data-driven, multimodal structural (DTI, VBM) and functional (fcMRI) imaging approach might be able to detect superregional, system-level alterations in HFA as it does not limit the analysis to predefined regions, tissue types or functional systems.

Diffusion tensor imaging (DTI) is based on the measurement of diffusion properties of water molecules [14]. It relies on the principle that diffusion occurs unrestricted in all directions in cerebrospinal fluid (isotropic), whereas in the white matter diffusion is restricted axially and increased parallel to fiber bundles (anisotropy). Fractional anisotropy (FA) can therefore serve as an indirect measure reflecting the underlying structural integrity of fiber tracts [15], where decreased FA values can reflect diminished myelination, reduced amount of axons, decreased axonal diameter and altered axonal interspaces in fiber tracts or bundles [16]. FA decrease in HFA has been reported in the corpus callosum [17], in white matter tracts in the temporal lobe [18], as well as in white matter pathways integrating frontal, temporal, and occipital structures involved in socio-emotional processing [19].

Voxel based morphometry (VBM) is a complementary approach to investigate structural brain abnormalities by assessing macroscopic volumetric changes of gray and white matter brain tissue. Structural neuroimaging studies in HFA have reported decreased white matter [3] and gray matter [20], [21] volumes, mainly in the frontal and temporal lobes.

Task dependent functional magnetic resonance imaging (fMRI) studies in HFA have shown various alterations [22], e.g. in selective attention [23] and executive function [24]. Owing to the heterogeneity of task paradigms final conclusions have been difficult to obtain, although many lines of investigation point towards cortical under-connectivity of brain regions activated by language processing [25], [26], and executive functioning [27], amongst others.

Functional connectivity magnetic resonance imaging (FcMRI) is an approach that allows for the detection of functional brain networks without task constraints. Functional connectivity has been defined as the temporal correlation of a neurophysiologic index measured in different brain areas [28]. In the context of resting state fcMRI the neurophysiologic index is the blood-oxygenation dependent (BOLD) signal, which is known to display low-frequency spontaneous fluctuations in the resting brain [29]. These signal fluctuations are spatially independent and show temporal correlation across largely separated cortical areas thereby forming functionally plausible networks [30]. Comprising mono- and polysynaptic functional connections, fcMRI is an indirect yet strongly associated measure of anatomical connections. Resting state fMRI studies in HFA patients have reported predominately decreased connectivity of various brain regions, like the medial prefrontal cortex [31], the right superior frontal gyrus [32], and the bilateral temporal lobe.

In summary the reported VBM, DTI and fcMRI findings indicate that individuals with HFA display alterations of gray matter volume, fractional anisotropy and functional connection strength, and that these alterations predominantly affect frontal and temporal association cortices.

We hypothesized that structural and functional alterations in HFA, although complex and widely distributed, affect similar or overlapping brain regions. We furthermore expected that these convergent structural and functional alterations are more likely to be located in multimodal association cortex areas rather than in unimodal primary sensory or motor cortex regions. To test these hypotheses, this study combined structural (DTI, VBM) and functional (fcMRI) data-driven imaging approaches.

## Materials and Methods

### Participants

All participants gave written consent before participating. The study was approved by the institutional review board of Ludwig-Maximilians University Munich and complies with the declaration of Helsinki. HFA participants were recruited from the outpatient clinic of the Department of Psychiatry and Psychotherapy, Ludwig-Maximilians University Munich. Healthy controls were recruited by advertisement and word of mouth. For subject characteristics see Table 1. DTI, VBM and resting state fcMRI data were obtained from 12 HFA participants that were diagnosed according to ICD-10 criteria (F.84.5) and 12 age- and gender matched healthy controls (HC). Exclusion criteria comprised history of major psychiatric disorders (e.g. depression, psychosis), seizure, head injury, toxic exposure and the evidence of genetic, metabolic, or infectious disorders. Individuals with secondary autism related to a specific etiology such as tuberous sclerosis or Fragile X syndrome were excluded.

**Table 1:** Demographic and Clinical Characteristics of the Participants.

	Control (n = 12, 4 female)		HFA (n = 12, 3 female)	
	Mean	SD	Mean	SD
Age (y)	33.3	9.0	35.5	11.4
AQ score^a^, sum	5.7	3.9	27.8	3.2
AQ score^a^, subscale social interaction	1.87	1.4	9.3	2.1
AQ score^a^, subscale fantasy	1.67	1.9	9.7	1.5
AQ score^a^, subscale communication	2.3	1.1	8.8	1.1
Full scale IQ^b^	110.8	14.4	111.3	13.4
Verbal scale IQ^c^	112.6	12.1	114.3	11.4

a Measured with the Autism Quotient questionnaire [35].

b Measured with the Standard Progressive Matrices (Raven, 1960).

c Measured using a vocabulary test [33] that is comparable to the Adult North American Reading Test [34].

### Diagnostic criteria and neuropsychological testing

All participants had a full scale IQ >85. The language-based IQ of HFA and HC individuals did not differ significantly (p = 0.70). The language based IQ was estimated using a vocabulary test [33] that is comparable to the English National Adult Reading Test [34]. In addition to expert clinical evaluation autistic traits were quantified by administration of the Autism Quotient Questionnaire (AQ) [35], [36]. AQ has been clinically validated [37] and widely used in autism studies [38], [39], It discriminated the two study groups significantly (p≤ 0.0001), with all HFA individuals ranging above the cut-off (≥17) for HFA [37], compared to none within the control group.

Two additional psychological tests were applied to the HFA group exclusively. The first one was the Freiburg Personality Inventory (FPI) which is a psychological test to assess personality traits [40]. It is comparable to the Eysenck Personality Questionnaire [41]. It comprises 138 items that are compiled into 12 scales: satisfaction with oneself, social orientation, need for achievement, shyness, irritability, aggressiveness, demandedness, physical troubles, health sorrows, openness, extraversion, and emotionality [40]. The second test is a questionnaire aiming at the assessment of empathy and appropriate social behaviour (QEAS). This questionnaire includes descriptions of 15 situations that can occur in daily social life that have to be commented by the participant [42].

### MRI data acquisition

Data acquisition was performed at a magnetic field strength of 3.0 Tesla (Magnetom VERIO, Siemens, Erlangen, Germany). DTI was performed using a singleshot spin-echo sequence and SENSE parallel imaging (undersampling factor of 3). Diffusion-weighted images were acquired in 20 non-collinear diffusion-encoding directions with multiple diffusion weighting b-values (b = 1000 s/mm2 and b = 0). Data acquisition parameters included the following: 25 contiguous (no-gap) 4.0 mm-thick axial slices with an acquisition matrix of 128×128 over a FOV of 230 mm, repetition time (TR)  =  9300 ms, echo time (TE)  =  102 ms, and spatial resolution  =  1.8×1.8×4.0 mm. The DTI sequence was repeated three times and averaged to obtain stable diffusion parameters. For an anatomical reference and the VBM analysis a high-resolution isotropic Magnetization Prepared Rapid Gradient Echo (MPRAGE) sequence was acquired (TE  =  7.6 ms, TR  =  14 ms, flip angle  =  20°, spatial resolution 0.8×0.8×0.8 mm, 160 slices). During the 6-minute resting state functional experiment participants were instructed to minimize head movement and stay awake with their eyes closed. Data was acquired using T2*-weighted echo planar imaging (TE  =  30 ms, TR  =  3000 ms, flip angle  =  90°, spatial resolution 3×3×4 mm, 36 slices, 120 volumes). All high-resolution T1 MPRAGE images were quality controlled by a board certified radiologist to detect potential artifacts such as ringing due to motion, spiking or signal loss. The imaging raw data illustrated in this study has been made publically available through the ABIDE (Autism Brain Imaging Data Exchange) consortium and can be downloaded here: http://fcon_1000.projects.nitrc.org/indi/abide/.

### DTI analysis

To investigate between group differences in FA values, we analyzed DTI data using the tract-based spatial statistics (TBSS) approach implemented in FSL 4.16 (Functional Magnetic Resonance Imaging of the Brain (FMRIB) Software Library, Oxford, UK). Images were corrected for eddy currents due to changing gradient fields and head motions [43]. The principal diffusion direction was established and the FA was calculated voxel-wise. To improve data quality, the FA maps of each subject were created using the averaged data of three DTI acquisitions. Brain masks were created using the Brain Extraction Tool (BET) [43]. FA data of all participants were aligned to a common space using the FMRIB58 FA standard space image of TBSS [44]. A mean FA image and a mean FA skeleton were created. FA values of each subject were projected onto the mean FA skeleton. Group differences in FA values were determined using Randomise version 2.6 (Permutation-based nonparametric testing, 5000 permutations) [45]. Results were corrected for multiple comparisons using threshold-free cluster enhancement for group contrasts.

### VBM analysis

Structural data analysis was performed using the VBM tool implemented in FSL 4.16. Structural images were brain-extracted using BET [43]. Tissue-type segmentation was carried out using FMRIB's Automated Segmentation Tool (FAST) [46]. The resulting gray matter partial volume images were normalized to MNI152 standard space (Montreal Neurological Institute, Montreal, Canada) using an initial affine (FMRIB's Linear Image Registration Tool, FLIRT) [47] and a subsequent nonlinear registration (FMRIB's Non-Linear Image Registration Tool, FNIRT) [48], [49]. The resulting warped gray matter images were averaged to create a study-specific template and the native gray matter images were then non-linearly re-registered to this template. To correct for local expansion or contraction the registered partial volume images were divided into the Jacobian of the warp fields (modulation). The modulated normalized gray matter images were then smoothed with an isotropic Gaussian kernel with a sigma of 4 mm. Each individual’s total gray matter was used as a covariate. Group differences in gray matter volume were determined using Randomise version 2.6 (Permutation-based nonparametric testing, 5000 permutations) [45]. We applied a statistical threshold with family-wise error rate (threshold-free cluster enhancement) corrected for multiple comparisons [50].

### Functional connectivity analysis

All data analysis steps were performed using FSL 4.16 and Analyses of Functional Images (AFNI, http://afni.nimh.nih.gov/afni). The first 5 functional scans of each session were discarded. We used the FMRI Expert Analysis Tool (FEAT) [51] for preprocessing of the fMRI scans. Motion correction was performed using FMRIB's Linear Image Registration Tool (MCFLIRT) [47].

Head motion has recently been shown to differentially impact functional connectivity measures [52]. As head motion may be higher in patients than in healthy controls, these motion effects can be mistaken as neuronal group effects [52]. To assess this potential confounding factor, the mean head motion of each subject, which represents the mean absolute displacement (in mm) of each brain volume as compared to the previous volume, was estimated from the translation parameters in x, y and z direction across all time points. Group comparison was performed using a two-sample t-test.

The skull was removed using BET [43] followed by a spatial smoothing using a 5-mm full width half maximum Gaussian kernel with high-pass temporal filtering (Gaussian-weighted least squares straight line fitting with sigma  =  50s). Individual high-resolution T1-weighted images were processed using AFNI. Registration to the high-resolution T1 and MNI-152 standard space template was carried out using FLIRT [47]. Preprocessed 4D data sets were re-sampled to 2 mm isotropic voxels in the following group analysis steps. Independent component analysis (ICA) was performed on all resting state runs using the Multivariate Exploratory Linear Optimized Decomposition into Independent Components (MELODIC) software 3.10 [53] in combination with a validated dual-regression approach [54], [55], [56]. The algorithm was set to automatically estimate the number of resulting components (resting state networks). The resulting eight group network maps were thresholded at an average z-score 2.3<z<10. Two group maps showed functionally implausible patterns likely reflecting non-neural signal and were excluded from further analysis. The remaining six networks served as „networks of interest“ for whole brain correlation analysis. This correlation analysis included voxels inside and outside the respective network. Therefore group differences in correlation strength could be found inside and outside the respective network. To perform voxel-wise comparison within and between subjects, probabilistic ICA [51] was applied to all individual, preprocessed data sets. We used Randomise 2.6 (permutation-based nonparametric inference, 5000 permutations) to determine voxel-wise nonparametric between group contrasts [45] for all eight networks. For each network we applied a statistical threshold with family-wise error rate (threshold-free cluster enhancement) corrected for multiple comparisons [50].

### Relationship between VBM and DTI measures

To test the relationship between measures of different imaging modalities an additional analysis called „linked independent component analysis for multimodal data fusion“ was performed. Linked ICA is a robust data fusion model that takes multi-modal data and characterizes inter-subject variability in terms of a set of multimodal components [57], [58]. This general model was configured to apply spatially concatenated ICA to obtain decomposition into spatial maps. As we applied the approach to structural modalities only, time course was not relevant for the algorithm. Therefore no tensorial ICA was applied. For further methodological details please refer to publications by Groves and colleagues [57], [58] who made their scripts publically available (http://fsl.fmrib.ox.ac.uk/fsl/fslwiki/FLICA). Previously preprocessed DTI and VBM files of HFA participants and controls served as the input. The algorithm was set to decompose the multimodal input into eight co-varying components. The resulting pseudo-z-scored component maps were normalized to values between -8 and 8, analogous to Groves and colleagues [57]. The images were then color-coded according to significance, with red-yellow showing larger values for subjects with positive weights (healthy controls) than subjects with negative weights (HFA).

### Correlation between imaging results and psychological testing

Psychological test results were correlated with FA, VBM and functional connectivity values within regions showing the most significant differences between HFA and HC. In order to maintain statistical power, we limited the correlation analysis to two out of twelve subscales of the FPI (Freiburg personality inventory, German personality test), namely emotionality and social interaction. These test items were chosen because alterations in these domains represent core features of HFA, rather than other subscales like aggressive behavior or health sorrows. The QEAS (questionnaire to estimate autistic symptoms) only consisted of 2 subscales, namely empathy and adequate social behavior, both of which were tested for correlation with imaging measures. In addition, we tested all subscales (social interaction, fantasy, and communication) and the sum of the Autism Quotient Questionnaire. Within each modality the significance of the Pearson product moment correlation results was Bonferroni-corrected for multiple testing (8 psychological test items in total). To correlate functional connectivity results we defined one region of interest (ROI) in the left STG based on the statistical group difference map obtained during fcMRI analysis and calculated the connection strength to the DMN. To correlate VBM measures, individual gray matter volume was summed up in a ROI in the left MTG, based on the VBM group difference map obtained previously. To correlate DTI measures we defined two regions of interest (ROI) based on the statistical group difference map obtained during DTI analysis. ROIs had a diameter of 16 mm around the peak voxel on the FA skeleton. FA values for each HFA subject were averaged within each of these two ROIs and correlated to psychological test results.

## Results

### DTI

**Figure 1:**
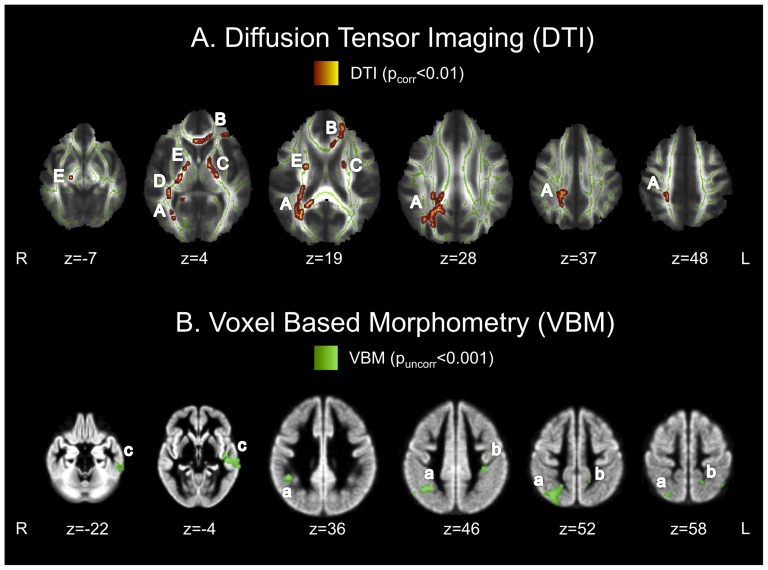
Structural between group differences. (A) The top row shows clusters of decreased FA values in high functioning autism (HFA) participants compared to healthy controls (HC) (p_corr_≤0.01). (B) The bottom row shows clusters of decreased gray matter volume in HFA compared to HC (p_uncorr_≤0.001). Clusters d-h are not displayed in the Figure but shown in Table 2. Indices A–E and a–c correspond to cluster indices in Table 2. Brains are displayed in radiologic convention (the right side of the brain Figure corresponds to the left hemisphere and vice versa).

At p_corr_≤0.01, DTI analysis revealed three main clusters of significantly reduced FA values in HFA when compared to HC: HFA individuals displayed lower FA values in a right-sided cluster reaching from the splenium of the corpus callosum into the superior longitudinal fasciculus within the parietal and temporal lobe and into the lateral occipital cortex (Table 2 and Figure 1, cluster A). A second cluster of reduced FA values in HFA participants was found in the anterior portion of the corpus callosum, reaching into the left anterior cingulate cortex and the left middle frontal cortex (Table 2 and Figure 1, cluster B). A further cluster of reduced FA values was detected within the corticospinal tract bilaterally (Table 2 and Figure 1, clusters C–E). No clusters of increased FA values in the patient group were detected.

**Table 2:** Structural differences between HFA individuals and healthy controls (HC>HFA), group differences in FA values derived from DTI analysis (clusters A–E, also see Figure 1) and group differences (HC>HFA) in gray matter volume (clusters a–j, also see Figure 1) derived from VBM analysis.

Cluster	Region	Hemi-sphere	No. of Voxels	p-value	Coordinates^a^ (X Y Z)		
	**DTI HC>HFA p_corr_≤ 0.01**						
A	Splenium corpus callosum, Superior longitudinal fascicle, Inferior longitudinal fascicle, TPJ, CG, SPL, LOL, STG, MTG, AG	R	2026	0.006	21	–45	19
B	Anterior corpus callosum, MFG, ACC	L	596	0.008	–3	26	1
B	Frontal lobe white matter, IFG	L	122	0.008	–33	37	–2
C	Posterior limb of internal capsule (anterior portion including corticospinal tract)	L	488	0.008	–12	–5	–3
D	Corticospinal tract	R	184	0.008	19	–17	–7
E	Posterior limb of internal capsule (anterior portion including corticospinal tract)	R	52	0.01	22	–8	12
	**VBM HC>HFA p_corr_≤0.1**						
c	MTG, ITG	L	148	0.072	–48	–22	–2
	**VBM HC>HFA p_uncorr_≤0.001**						
a	SPL, SMG, AG, TL, LOL, TPJ	R	540	≤0.0001	44	–42	34
b	SPL, SMG, AG,TL, LOL, TPJ	R	145	≤0.0001	22	–60	52
c	MTG, ITG, FuC	L	1016	≤0.0001	–64	–38	–26
	**VBM HC>HFA p_unpcorr_≤ 0.01**						
a	SPL, SMG, AG, TL, LOL, TPJ, (SLF)	R	4392	≤0.001	44	–42	34
b	SPL, SMG, AG, TL, LOL, TPJ, (SLF)	L	6219	≤0.001	–46	–48	6
c	MTG, ITG	L	5271	≤0.001	–64	–38	–26
d	MTG, ITG	R	808	0.001	70	–24	–6
e	IFG, OFC, FP	R	2472	≤0.001	36	40	40
f	IFG, OFC, FP	L	826	0.001	–50	46	12
g	FP	L	534	≤0.001	–12	38	–30
h	MFG	L	202	0.003	–30	16	28
i	MPFC, CG	BL	1720	≤0.001	–2	66	28
j	Insula	L	164	0.001	–42	–2	20

Abbreviations: ACC = anterior cingulated cortex, AG = angular gyrus, CG = cingulated gyrus, FP = frontal pole, IFG = inferior frontal gyrus, ITG = inferior temporal gyrus, LOL = lateral occipital lobe, MFG = middle frontal gyrus, MPFC = medial prefrontal cortex, MTG = medial temporal gyrus, OFC = orbito-frontal cortex, SMG = supramarginal gyrus, SPL = superior parietal lobe, STG = superior temporal gyrus, TL = temporal lobe, TPJ = temporo-parietal junction;

a Coordinates are given in MNI152 standard space.

### VBM

VBM analysis revealed bilateral clusters of reduced gray matter volumes in the frontal, temporal and parietal lobe for HFA compared to HC. HFA individuals showed a trend of reduced gray matter volume in the left medial and inferior temporal gyrus (p_corr_≤0.1). At an uncorrected p-value (p_uncorr_≤0.001) clusters of reduced gray matter volume of HFA were detected within the bilateral temporo-parietal junction area (Table 1 and Figure 1, clusters a and b), the bilateral temporal lobe (Table 2 and Figure 1, cluster c and d) and the bilateral inferior frontal gyrus including the left frontal pole and left middle frontal gyrus (Table 2, clusters e–h). The bilateral medial frontal and paracingulate gyrus also displayed reduced gray matter volumes in the patient group (Table 2, cluster i). No clusters of increased gray matter volume in the patient group were detected.

### Functional connectivity

**Figure 2:**
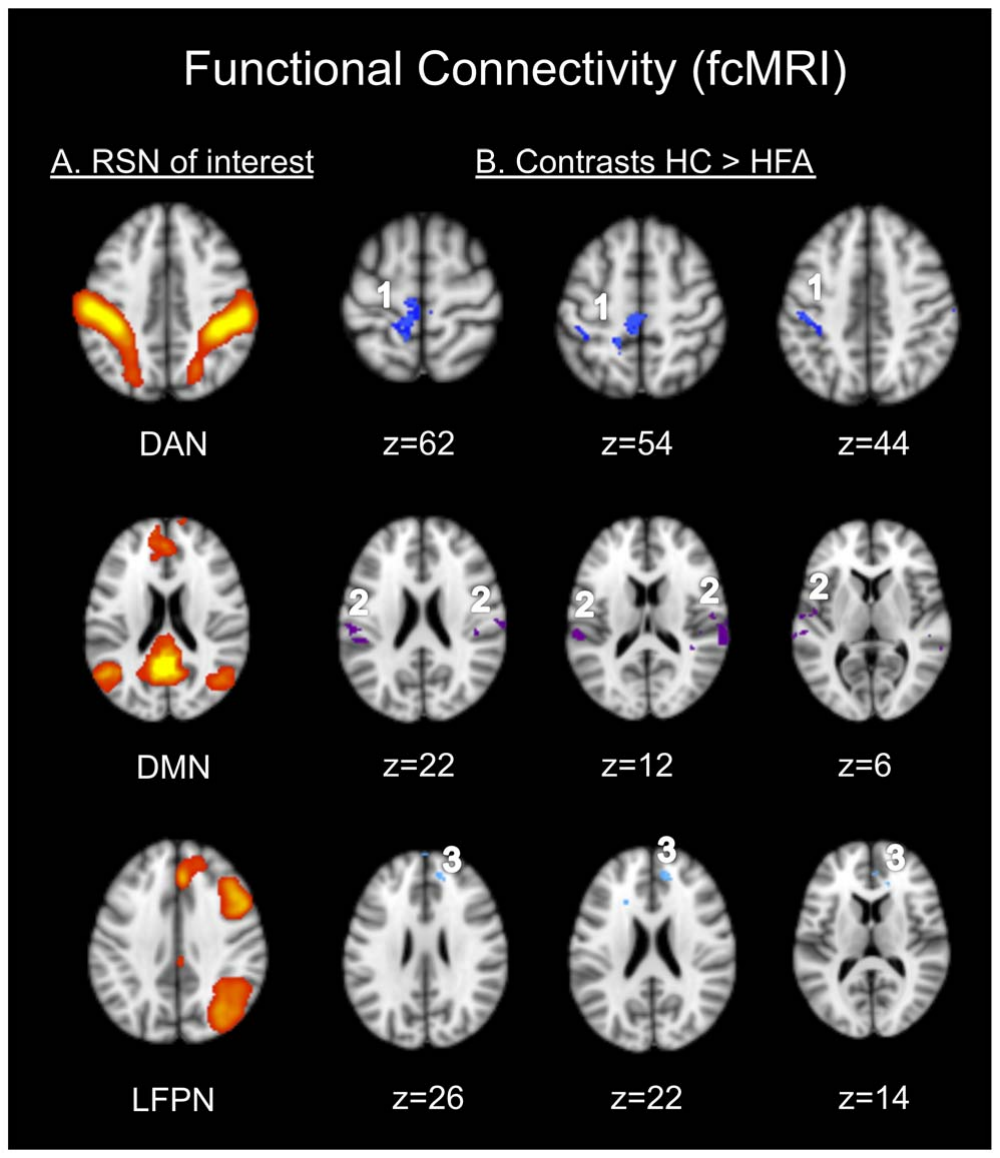
Functional connectivity between group differences. (A) Resting state networks (RSN) of interest, identified by group-ICA of resting state fMRI data and subjected to analysis of functional connectivity differences between high functioning autism (HFA) participants and healthy controls (HC). Three RSNs, namely the DAN (dorsal attention network), the DMN (default mode network) and the LFPN (left-lateralized fronto-parietal network) exhibited clusters of decreased functional connectivity in HFA as compared to HC (B). Indices 1–3 correspond to cluster indices in Table 2. For visualization purposes group differences are displayed at p_uncorr_≤0.001, corrected significance levels can be found in Table 3. Brains are displayed in radiologic convention (the right side of the brain Figure corresponds to the left hemisphere and vice versa).

Two out of eight networks were excluded from further analyses because they clearly represented non-neural signal related to motion and/or respiratory/circulatory artifacts, as has been previously described [30], [56]. The remaining six components constituted neurophysiologically meaningful networks that have been observed in previous ICA-based studies of intrinsic functional connectivity and have been proven to be reliable [30], [56]. These networks included the dorsal attention network (DAN), the default mode network (DMN), the left and right fronto-parietal networks (LFPN, RFPN), the frontal executive control network (FECN) and the sensory-motor-network (SMN). The between group comparison of head motion, measured by the mean relative displacement (in mm), revealed no significant group differences (HFA: 0.0234±0.0087, HC: 0.0215±0.0091, p = 0.6191). Significant group differences in functional connectivity at p_corr_≤0.05 were found for the dorsal attention network where HFA individuals showed lower connectivity to a cluster including the right precentral gyrus, which reached into the right parietal lobe (Table 3 and Figure 2, cluster 1). At p_uncorr_≤0.0005 this cluster reached further into the right parietal and precuneal cortex. The HFA also showed significantly reduced connectivity between the DMN and the left superior temporal gyrus at p_corr_≤0.05 and the bilateral superior temporal gyri at p_corr_≤0.1 (Table 3 and Figure 2, cluster 2). A third cluster of reduced connectivity emerged from the LFPN comparison. Here, HFA participants displayed reduced connectivity to the left anterior cingulate cortex (ACC) and to the medial prefrontal cortex (Table 3 and Figure 2, cluster 3). No increased functional connectivity in the patient group could be detected. Group comparison of the connectivity profiles of the RFPN, the FECN and the SMN did not reveal any significant group differences.

**Table 3:** Brain regions showing clusters of significant differences in RSN connectivity between HFA probands and controls (HC>HFA).

RSN of Interest	Cluster (Figure 2 and 3)	Region	Hemi- sphere	p-value	voxel	Coordinates^a^ (X Y Z)		
**Dorsal Attention Network**	1	PreCG, PCC	R	p_corr_≤0.05	73	6	–26	52
	1	PreCG, PoCG, PCU, PCC	R	p_corr_≤0.1	166	6	26	52
		SPL, PoCG	R		13	40	–30	46
	1	SPL, PreCG,PoCG, PCC, PCU	R	p_uncorr_≤0.0005	446	18	–42	50
	1	SPL, IPL, PoCG, SMG	R		149	36	–36	34
**Default Mode Network**								
	2	STG	L	p_corr_≤0.05	4	–64	–34	10
	2	STG	L	p_corr_≤0.1	105	–64	–34	10
	2	STG (planum temporale)	R		39	60	–28	12
	2	Parietal operculum (planum temporale)	R		23	58	–22	20
	2	STG	L	p_uncorr_≤0.0005	434	–60	–38	–10
	2	STG	R		164	60	–26	4
**Left**	3	MPFC/ACC	L	p_corr_≤0.1	2	0	44	16
**Frontoparietal**	3	MPFC/ACC	L	p_uncorr_≤0.0005	60	–10	44	18
**Network**								

Abbreviations: ACC = anterior cingulated gyrus, IPL = inferior parietal lobe, MPFC = medial prefrontal cortex, PCC = posterior cingulated gyrus, PCU = precuneus, PoCG = postcentral gyrus, PreCG = precental gyrus, RSN = resting state network, SMG = supramarginal gyrus, SPL = superior parietal lobe, STG = superior temporal gyrus;

a Coordinates are given in MNI152 standard space.

### Convergent sites of structural and functional differences

**Figure 3:**
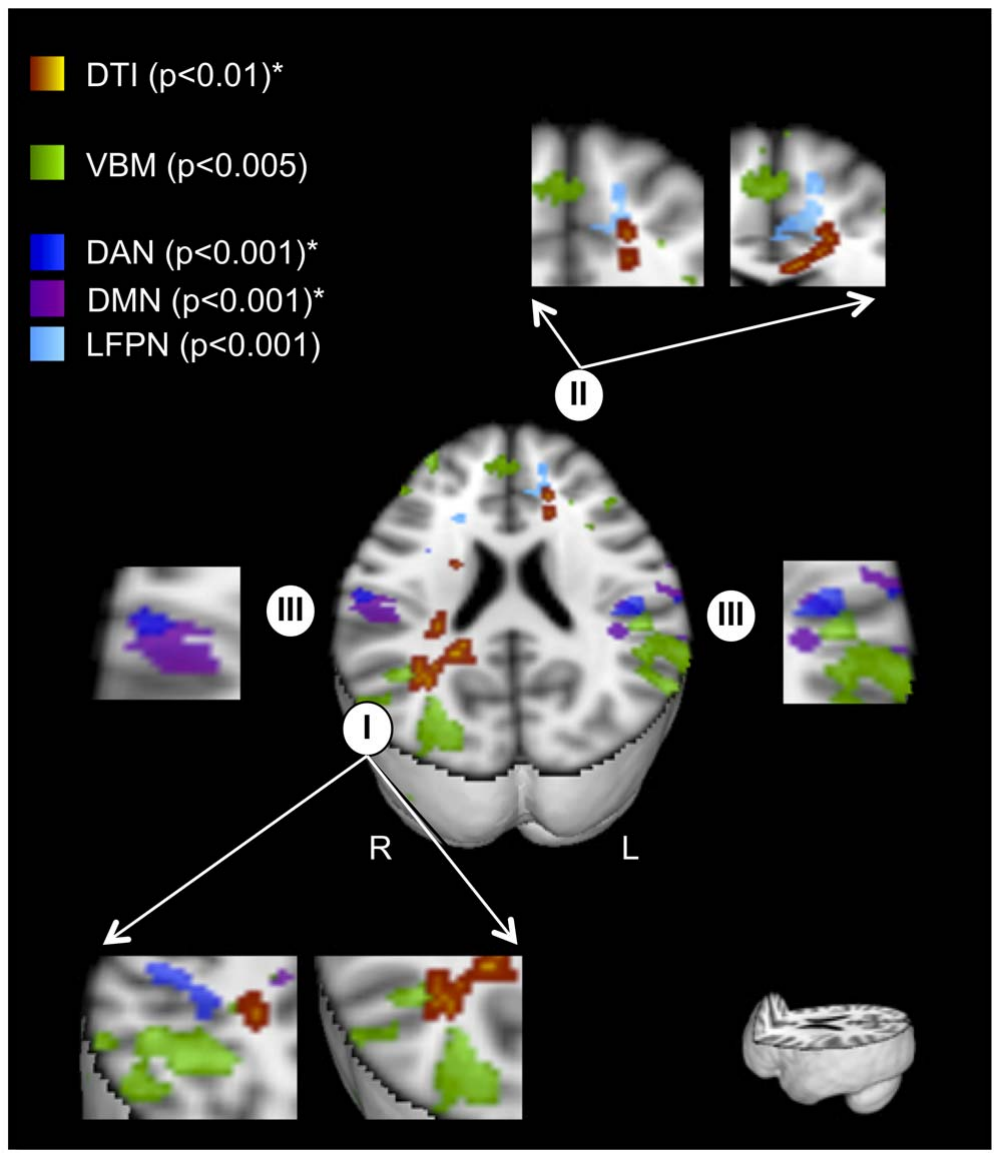
Localization of common sites of structural and functional alterations in high functioning autism. (I) indicates the right temporo-parietal cluster and (II) indicates the left medial frontal cluster of convergent DTI, VBM and fcMRI findings in Asperger’s syndrome; (III) indicates a bilateral temporal cluster of concomitant fcMRI and VBM alterations in HFA participants. For visualization purposes VBM and fMRI group differences are displayed at p_uncorr_≤0.001, corrected significance levels can be found in Table 2 and 3 respectively. All DTI, VBM and fMRI results that survived multiple comparison correction are marked with an asterisk (*). Brains are displayed in radiologic convention (the right side of the brain Figure corresponds to the left hemisphere and vice versa).

We found three regions of combined structural and functional alterations (Figure 3, clusters I to III). The right temporo-parietal junction area and the left frontal lobe showed decreased FA values along with decreased functional connectivity of the DAN and the LFPN respectively and a trend towards decreased gray matter volume (Figure 3, clusters I and II). The superior temporal gyrus displayed significantly decreased functional DMN connectivity in HFA that was accompanied by the most prominent trend of gray matter volume decrease in the temporal lobe of HFA individuals (Figure 3, cluster III).

Linked independent component analysis for multimodal data fusion further revealed eight components of co-varying VBM and/or DTI measures, specifically six components of co-varying VBM measures, one component of co-varying DTI measures and one composite component of co-varying VBM and DTI measures This composite component was contributed by DTI and VBM measures with a relative weight of DTI of about 12% and a relative weight of VBM of about 88%. The DTI and VBM values of this component co-varied in multiple brain regions, including the right temporo-parietal junction area and the left frontal lobe (see Figure S1). The DTI and VBM maps of this component were color-coded according to significance (z-statistic), with red-yellow showing larger values for subjects with positive weights (healthy controls) than subjects with negative weights (HFA). To show the effects within regions that show a disease effect in HFA, DTI and VBM results shown in Figure 1 were applied as masks to the DTI and VBM component maps respectively. These masks were applied after analysis for visualization purposes only.

### Correlation between imaging results and psychological testing

**Figure 4:**
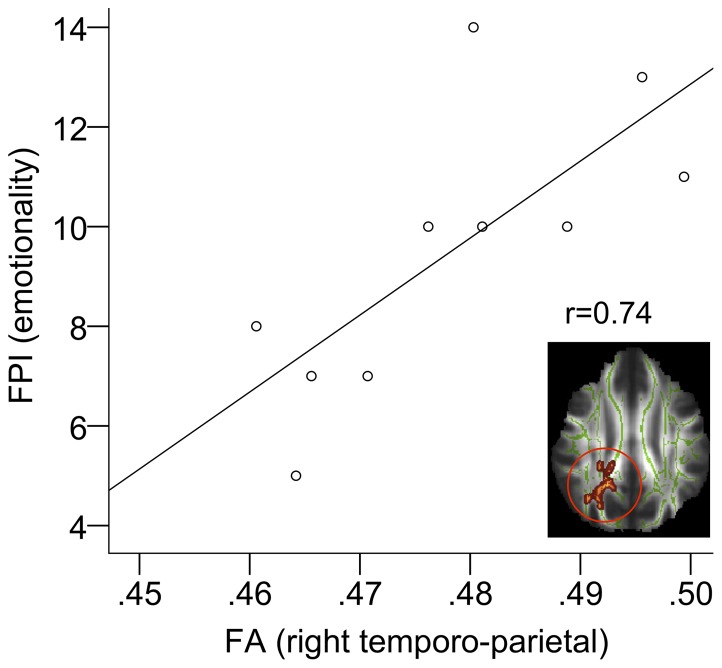
Correlation between DTI results and psychological test data. This plot shows the correlation between test results of the FPI (Freiburger Personality Inventory) test, subscale emotionality, and DTI results. Individual FA values within the white matter skeleton were averaged across the right temporo-parietal region.

Within HFA patients the only significant correlation was detected between FA values in the right temporo-parietal region (Table 2, cluster A) and FPI results (subscale emotionality, r = 0.74, p_corrected_<0.05, Figure 4). FA values in the right temporo-parietal junction did not show any significant correlation to the AQ sum score or subscales (social interaction, communication and fantasy), to the FPI subscale empathy, or to the QEAS subscales empathy and adequate behavior. There was no significant correlation between psychological test data and FA values in the left frontal cluster (Table 2), VBM measures or fcMRI measures.

## Discussion

The present study combined structural (DTI, VBM) and functional (fcMRI) data-driven imaging approaches to detect subtle, complex and widely distributed brain alterations in individuals with HFA and to investigate whether these alterations affect similar brain regions across modalities. We found three regions of convergent structural and functional alterations in HFA, all of which were located in higher order association cortex. The two regions displaying the most prominent FA value decrease, namely the right temporo-parietal junction area and the left frontal lobe showed concomitant functional connectivity decrease to the DAN and the LFPN respectively and a trend towards decreased gray matter volume. Interestingly, DTI alterations in these two main clusters seemed to be located down the neuronal stream to VBM changes. A formal testing of the relation between these structural properties revealed that DTI and VBM measures in these two clusters co-varied across participants, meaning that high FA values coincided with high gray matter volume and vice versa. In a third cluster of convergent structural and functional alterations decreased functional DMN connectivity in the bilateral superior temporal gyrus was accompanied by a trend towards gray matter volume decrease in the temporal lobe of individuals with HFA.

The functional and structural differences between HFA and HC individuals in the right temporo-parietal cortex found in this study are in line with previously reported abnormalities of parietal lobe anatomy in terms of alterations in FA-properties [59], and white matter volume [3] as well as decreased functional connectivity in the bilateral temporo-parietal area in HFA [27], [60], [61]. Temporo-parietal regions are linked to emotional processing [62], theory of mind [63], and empathy [64]. A recent fMRI experiment focusing on reflection-processes on self and others was able to relate altered activation of the right temporo-parietal junction to social symptom severity in HFA [65]. Emotionality is typically affected by autism pathology [66] and its neural correlates have recently been shown to be affected by ASD risk genes that are associated with alterations in circuits that mediate socio-emotional processing [67]. In this study, the extent of white matter integrity loss in the temporo-parietal junction area, estimated by FA decrease, was found to correlate with the extent of impairment in emotional processing. Moreover, measures of structural integrity (FA and VBM) in this region could be shown to co-vary, pointing towards a possible pathophysiologic relationship between gray and white matter alterations in this region in HFA. In summary our results indicate that structural and functional alterations in the area of the temporo-parietal junction may account for the deficit in emotion processing which is one of the pathologic core features of HFA.

The temporal lobe is another region known to be affected by autism pathology [18]. The results of this study revealed a loss of gray matter integrity within the superior temporal gyrus in HFA and a disruption in functional connectivity between the bilateral superior temporal lobe and the default mode network. These findings are in line with the notion that the bilateral superior temporal gyrus plays an important role in cognitive domains that are typically impaired in ASD such as social cognition [68], [69], social perception of biological movement [70] and theory of mind [63].

Convergent structural and functional alterations in HFA were also revealed in a mainly left-sided cluster including the prefrontal cortex, the ACC and the anterior corpus callosum. Differences in frontal lobe anatomy and function have previously been described in Asperger’s syndrome [71], [72] and have lead to the (frontal) underconnectivity theory of autism [25], [73]. PET and fMRI studies have described reduced activity in the ACC and ventromedial prefrontal cortex (VMPFC) during various tasks in HFA samples including Asperger’s syndrome [74], [75]. For instance, frontal lobe deficits have been linked to abnormalities in joint attention [76] and theory of mind [77]. In general, social functioning relies on multiple neural structures including the ACC and the VMPFC [69]. Accordingly, altered functional connectivity, activation and morphology within these regions might be associated with social impairment in ASD [78], [79].

The main sites of functional and structural pathology reported in the present study can be attributed to higher order association cortex while primary sensory and motor cortices show almost no significant differences in functional connectivity strength or structural integrity. Higher order association areas are located in the prefrontal, the superior temporal and in the inferior parietal cortex and are known to display similar cytoarchitectonic properties [80], [81], [82]. These multi-modal integration centers between internal and external components of the sensorium also show specific properties in fcMRI. For example, frontal, temporal and parietal association cortex has recently been shown to display much higher inter-individual variability in functional connectivity than primary sensor and motor cortex [83], rendering these regions more likely to display disease-relevant inter-individual differences. Furthermore these regions are known to exhibit especially high functional connectivity and are likely to connect functionally more specialized regions, thereby serving as “cortical hubs” [84]. It has been proposed that ASD is characterized by an impairment of higher-order multisensory integration [85] and information processing [86], which are crucial prerequisites for perception of, and flexible, meaningful and productive responses to, the (social) environment. This ability to integrate information across a variety of contexts (perception, attention, language, semantics) is impaired in autism and has been referred to as the ‘‘weak central coherence’’ theory [87], [88]. Given that such higher-order multisensory processing relies on the rapid exchange of information, putatively enabled by cortical hub regions, it can be speculated that the functional and structural changes in the regions reported in this study represent correlates of impaired multisensory integration in autism.

One limitation to our study is that no formal analysis of the relationship between functional connectivity and structural changes was performed. While the relation between DTI and VBM measurements had been successfully explored before [57], [58], the relationship between functional connectivity measures and structural measures is less straightforward. Even in large data sets of healthy subjects, such a relationship is hard to establish as "[...] strong functional connections commonly exist between regions with no direct structural connection, rendering the inference of structural connectivity from functional connectivity impractical" [89], also see Damoiseaux and Greicius 2009 [90]. We therefore restrained from exploring such a relationship in our relatively small data set. Another limitation to our study is the relatively small sample size that may have reduced statistical power. However, the statistical significance of DTI and functional connectivity results after correcting for multiple comparisons is remarkable given the whole brain data driven approach, which is even more prone to false negative results than any region of interest analysis. Although VBM results did not survive multiple comparison correction, a statistical trend could be detected. The exclusive focus on Asperger’s syndrome, a well-defined sub-entity of HFA, as opposed to the inclusion of a broad range of heterogeneous autism spectrum disorders, constitutes a valuable study sample. The results of the present study are exploratory and need to be confirmed in larger study samples. However, the convergence of findings across different imaging modalities, which in case of the right temporo-parietal area, are further supported by correlations with psychological test data, support the conclusion that the described regions do have implications in HFA and might serve the field for further hypothesis generation.

## Conclusion

In conclusion, the results of the present study indicate that brain alterations in HFA, although subtle and widely distributed, converge into common sites of structural and functional differences in higher order association cortex areas such as the temporo-parietal junction and the prefrontal cortex in individuals with high functioning autism. These findings support the long-standing hypothesis of autism as a disorder of impaired higher-order multisensory integration.

## Supporting Information

Figure S1 **Relation between DTI and VBM parameters.** To investigate the relationship between DTI and VBM variance, “linked independent component analysis for multimodal data fusion”, a data fusion model that characterizes inter-subject variability of multi-modal data in terms of a set of multi-modal components, was performed. Here we show one component where DTI and VBM parameters co-varied across participants. This component comprised multiple brain regions, including the left frontal cortex and right temporo-parietal cortex. The images are color-coded according to significance (z-statistic), with red-yellow showing larger values for subjects with positive weights (healthy controls). To show the effects within regions that show a disease effect in HFA, DTI and VBM results shown in Figure 1 were applied as masks for visualization purposes only.
(TIFF)
