# Supplementary figures and images for: Convergent Findings of Altered Functional and Structural Brain Connectivity in Individuals with High Functioning Autism: A Multimodal MRI Study
Source: PLoS One. 2013 Jun 18;8(6):e67329. doi: 10.1371/journal.pone.0067329 (PMC3688993; doi:10.1371/journal.pone.0067329)

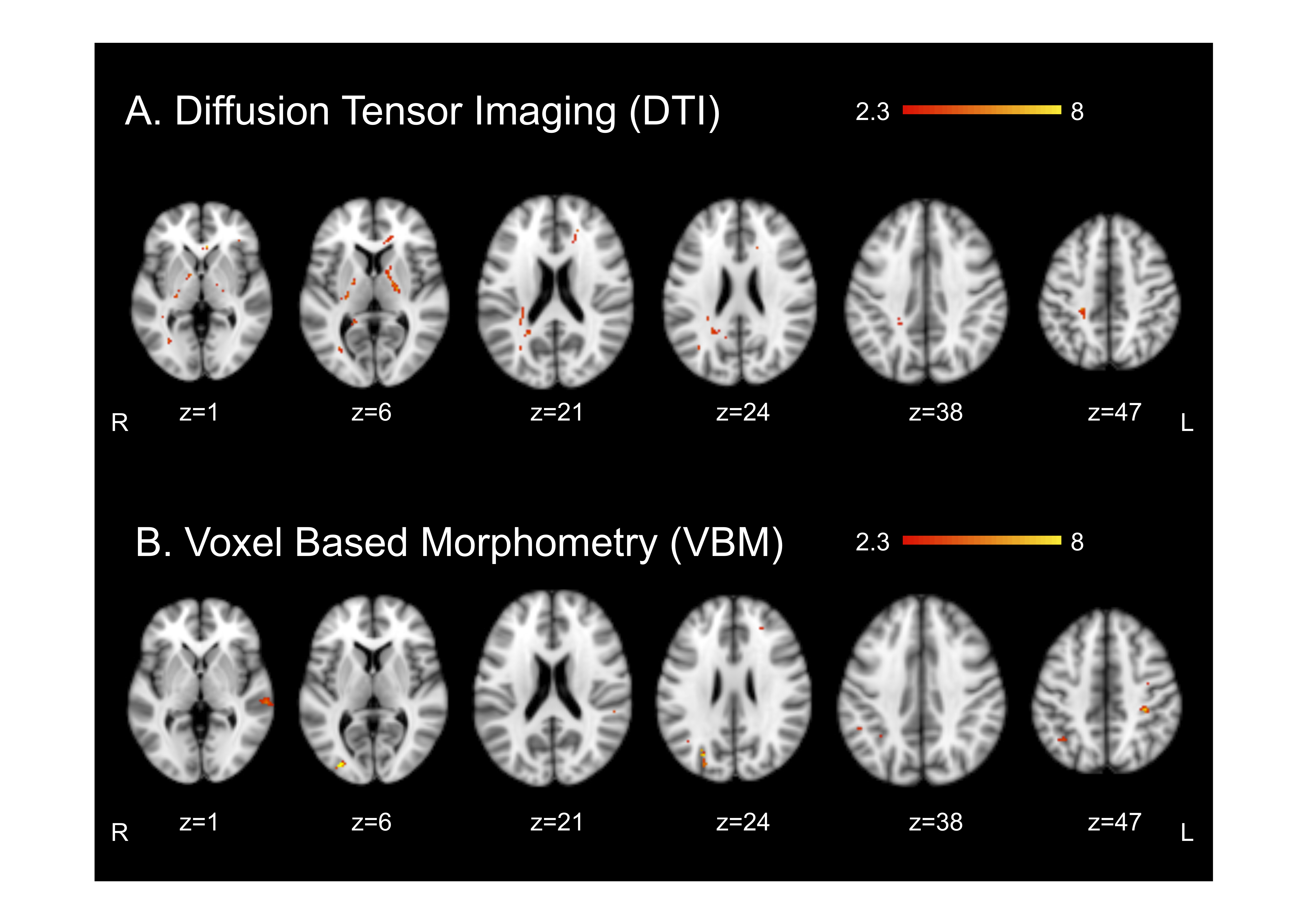

Supplement: Figure S1 — Relation between DTI and VBM parameters. To investigate the relationship between DTI and VBM variance, “linked independent component analysis for multimodal data fusion”, a data fusion model that characterizes inter-subject variability of multi-modal data in terms of a set of multi-modal components, was performed. Here we show one component where DTI and VBM parameters co-varied across participants. This component comprised multiple brain regions, including the left frontal cortex and right temporo-parietal cortex. The images are color-coded according to significance (z-statistic), with red-yellow showing larger values for subjects with positive weights (healthy controls). To show the effects within regions that show a disease effect in HFA, DTI and VBM results shown in Figure 1 were applied as masks for visualization purposes only. (TIFF) [file pone.0067329.s001.tif]
